# The Psychological Impact of Participation in Victim-Offender Mediation on Offenders: Evidence for Increased Compunction and Victim Empathy

**DOI:** 10.3389/fpsyg.2021.812629

**Published:** 2022-01-28

**Authors:** Jiska Jonas, Sven Zebel, Jacques Claessen, Hans Nelen

**Affiliations:** ^1^Department of Psychology of Conflict, Risk and Safety, Faculty of Behavioural, Management and Social Sciences, University of Twente, Enschede, Netherlands; ^2^Criminal Law and Criminology, Faculty of Law, Maastricht University, Maastricht, Netherlands; ^3^Dutch Private Law, Faculty of Law, Vrije Universiteit Amsterdam, Amsterdam, Netherlands

**Keywords:** restorative justice, victim-offender mediation, psychological impact, offender, reoffending

## Abstract

Participation in victim-offender mediation (VOM) can reduce the risk of reoffending. However, relatively little is known about how VOM affects the intermediate psychological changes underlying this effect. It was hypothesized that VOM increases feelings of responsibility, guilt, and shame among offenders as well as empathy toward the victim. It was also expected that VOM leads to feelings of moral failure among offenders, increasing their intention to desist, and improving their relation with the victim, relatives, and community. Lastly, it was hypothesized that offenders may experience reduced rejection, concerns about condemnation, threat to their social moral identity, and victim blame following VOM. To this end, we compared psychological changes in offenders who participated in a VOM program in the Netherlands with those of offenders who were willing to but did not participate (total *N* = 86). A quasi-experimental, pre- and postmeasure research design was used to compare these groups. Our findings tentatively suggest that offenders who participate in VOM have more responsibility-taking and victim empathy, feel more guilt and shame, and experience higher moral failure than offenders who do not participate in VOM do. Offenders also reported feeling significantly less awkward about meeting the victim again after VOM. Future research should address how and to what degree these psychological changes translate into a lower risk of reoffending.

## Introduction

The practice of (and research into) restorative justice continues to grow (D’Souza and L’Hoiry, 2019). The key component of restorative justice is giving the offense back to the main involved parties of a crime: victims, offenders, and the community. Instead of aiming to punish offenders, restorative justice focuses on what the involved parties need. In this way, attempts are made to resolve the harm that has been done, and offenders are encouraged to take responsibility. Facilitating and organizing a constructive dialogue between the parties is important to achieve these goals (Umbreit et al., 2004; Zehr, 2015; Claessen and Roelofs, 2020). This dialogue should give victims and offenders the opportunity to ask questions about the offense, explain the consequences of the offense, and come to a mutual agreement about how to repair the damage that has been inflicted.

Victim-offender mediation (VOM) is an example of a restorative justice program (Hansen and Umbreit, 2018). VOM is a dialogue-driven process, in which victims and offenders have the opportunity to communicate voluntarily with each other about the offense, in the presence of a trained mediator (Hansen and Umbreit, 2018). First, the mediator meets with the offender and the victim separately. When the mediator appraises that a constructive meeting between the offender and victim is possible and desired, a joined conversation may take place. If the parties want contact but do not want to meet face-to-face, other means of communication are possible, such as exchanging letters or exchanging messages *via* the mediator (shuttle mediation) (Zebel, 2012; Hansen and Umbreit, 2018; Claessen and Roelofs, 2020).

Evidence suggests that VOM can benefit both victims and offenders (e.g., Abrams et al., 2006; Saulnier and Sivasubramaniam, 2015; Jonas-van Dijk et al., 2020). Restorative justice often increases the satisfaction of victims and offenders compared with conventional criminal justice procedures without the option of restorative justice (Poulson and Elton, 2002; Meléndez, 2015). VOM can also reduce anger and fear in victims (Zebel, 2012) and gives offenders the chance to deal with their emotions by apologizing and showing regret (e.g., Choi, 2008; Lauwaert and Aertsen, 2016).

Restorative justice programs like VOM may also reduce the risk of reoffending. Although this is not the aim of restorative justice (Zehr, 2015), it is one of the most researched themes in relation to VOM. Multiple studies have concluded that offenders who participate in VOM have a lower risk of reoffending than offenders who did not participate in VOM do (Bergseth and Bouffard, 2013; Claessen et al., 2015; Jonas-van Dijk et al., 2020). However, some researchers are critical about the effects of VOM on reoffending. Jonas-van Dijk et al. (2020) have argued that reduced reoffending could be based on self-selection bias, since participation in mediation is voluntary. This means that offenders who are willing to participate in VOM might be less likely to reoffend than offenders who are not willing to participate are (Elbers et al., 2020). Nevertheless, Jonas-van Dijk et al. (2020) have shown that self-selection bias may partly but not completely explain reduced reoffending and that the VOM process itself is at least partly responsible.

If VOM can reduce reoffending, it is logical to assume that it incites psychological mechanisms that change the behavior of the offender. However, the psychological changes that underly this reduced reoffending after VOM remain undefined. To our knowledge, systematic quantitative research studies have not been conducted to answer this question. This article intends to fill this knowledge gap.

### Psychological Impact of Victim-Offender Mediation

Multiple qualitative studies have examined what happens during a VOM meeting and how this influences participants. Research indicates that talking to the victim can help offenders realize the impact of their crime and see the victim behind the offense (Choi et al., 2011). This can lead to stronger feelings of guilt and empathy (Abrams et al., 2006; Miller and Hefner, 2015; Meléndez, 2020a), which might lower the risk of reoffending (Tangney et al., 2014; Schalkwijk et al., 2016; Vaish et al., 2016). Empathy is often differentiated into cognitive and affective dimensions. The cognitive factor, perspective taking, describes the ability to put oneself in another person’s position and imagine their perspective. The affective factor, empathic concern, describes the ability to feel and understand the feelings of another person (Leith and Baumeister, 1998; De Corte et al., 2007). Based on these qualitative studies, it is expected that offenders who participated in VOM have stronger feelings of guilt, higher victim empathy, and more perspective taking than offenders who did not participate in VOM do.

An important part of VOM is discussing what happened, why the offender committed the offense, and how the offender can take responsibility (Pabsdorff et al., 2011). A central aim of restorative justice is to hold offenders accountable for their wrongdoings (Umbreit et al., 2004; Zehr, 2015; Claessen and Roelofs, 2020). Research suggests that this goal is achieved during VOM – offenders were held more accountable for their crimes during VOM meetings than during court procedures without VOM (Boriboonthana and Sangbuangamlum, 2013). Based on this, we expect that offenders who participated in VOM will report feeling more responsible for their offense than offenders who did not participate in VOM will. We are also interested in victim blaming because this might interfere with the ability of offenders to take responsibility for their actions – i.e., offenders who blame the victim might take less responsibility (Henning and Holdford, 2006). Since we expect offenders to take more responsibility after participating in VOM, we also expect reduced victim blaming among these offenders.

The relationship between the offender and the victim, relatives, and community can be restored by VOM. The victim and offender might find common ground during VOM and resolve their conflict (Meléndez, 2015). van Denderen et al. (2020) observed that VOM can restore the relationship between the victim and offender if they knew each other before the offense. Participating in VOM can also impress friends and relatives of the offender (Shapland et al., 2007), which we believe might help them to restore their relationships even though they are not part of the conversation. We therefore expect that offenders who participate in VOM will view their relation with the victim, relatives, and community as more positive after VOM than offenders who do not participate in VOM and are therefore less willing to restore this relationship will.

Another important factor that is often associated with restorative justice practices, is the experience of shame among offenders. The reintegrative shaming theory of Braithwaite (1989) describes two disapproving responses to offenders after a crime that might create shame: a stigmatizing or reintegrative response. When offenders are responded to in a stigmatizing manner, disapproval about the crime is disrespectful and the person is being labeled as an outcast. According to the societal reaction or labeling theory (Lemert, 1973), this labeling likely encourages the offender to show deviant behavior in the future. In other words, stigmatization may foster reoffence. When offenders are responded to in a reintegrative manner, disapproval is respectful and focuses on the crime rather than on the person. As a result, offenders are less likely to feel labeled or stigmatized as a criminal and are therefore less likely to reoffend (Braithwaite, 1989).

In a similar vein, Gausel et al. (2016) defined how people respond after committing a transgression. They postulated that a transgression can be appraised in two ways: either as a moral failure or as a risk to their social-moral image. Much like when the offender is responded to in a reintegrative manner, offenders who perceive their offense as a moral failure will be self-critical and understand that their behavior was not according to internalized rules and norms. This may lead to subjective feelings of shame, humiliation, and disgrace. Gausel et al. (2016) explained that the best way to repair this self-defect and deal with these feelings of shame is to restore the defect and the self. By apologizing and/or offering compensation to victims, offenders can show themselves and others that they are acting according to existing rules and norms.

However, when the transgression is considered a risk to their social-moral image, offenders might fear condemnation from others, which may lead to feelings of rejection. According to Gausel et al. (2016), offenders are likely to respond defensively to this perceived condemnation and rejection, which may manifest into avoidance and cover-up behaviors, such as not taking responsibility and attempts to justify or rationalize their behavior.

These theories and arguments underline the importance of treating offenders in a respectful manner during VOM to avoid impairing their social-moral image. Previous research has indicated that restorative justice programs are experienced as less stigmatizing and judgmental than traditional retributive justice procedures (Shapland et al., 2008; Lauwaert and Aertsen, 2016). It has also been argued that retributive punishments can increase recidivism because the official reaction (e.g., going to a court hearing, being treated as a suspect) facilitates labeling and stigmatization (Miethe et al., 2000). Taken together, it is expected that offenders who have participated in VOM will consider their behavior more immoral (a specific self-defect) and feel more ashamed about this than offenders who have not participated in VOM will. However, they will be less concerned about condemnation and experience less rejection. This could explain how participating in VOM might lower the risk of reoffending.

If offenders who participated in VOM feel less rejected than offenders who did not participate in VOM do, then their social moral identity may be less threatened after VOM. Shnabel and Nadler (2008, 2015) explained that an offenders’ social-moral image might be impaired after an offense. Every person has different social identities, with which they identify with other people and groups based on traits, family bonds, or other life experiences (Markus, 1977; Aquino and Reed, 2002). The moral-social identity is one such social identity (Aquino and Reed, 2002). When offenders are perceived as socially immoral (belonging to the immoral group of ‘criminals’), they might need to restore their moral-social identity to avoid social exclusion (Shnabel and Nadler, 2008, 2015). VOM may allow offenders to do this by giving them the opportunity to make amends and their victims the opportunity to show understanding and grant forgiveness. In this way, the victim confirms that the offender is not a criminal, reducing the risk of social exclusion. Thus, in line with reduced feelings of rejection after VOM, it is expected that offenders who have participated in VOM experience a lower threat to their social moral identity than offenders who did not participate in VOM do.

The last factor that we will consider is the motivation to desist from crime. Desistance is the process by which offenders detach themselves from their criminal behavior pattern (McNeill et al., 2012). Some scholars refer to desistance as a key turning point in the life of criminals (Laub and Sampson, 1993). Lauwaert and Aertsen (2016) concluded that mediation is not always a trigger for desistance, but can support a desistance process that is already underway. This indicates that mediation is not a turning point in itself, but rather a way to reinforce motivation to desist from crime. There may also be a difference in the motivation to desist between offenders who are willing to participate and offenders who are not willing to participate in VOM. Even if offenders have already started to desist, the VOM process probably supports and further reinforces this motivation (Lauwaert and Aertsen, 2016). Therefore, it is expected that offenders are more willing to desist from crime after VOM compared to offenders who did not participate in mediation.

**FIGURE 1:**
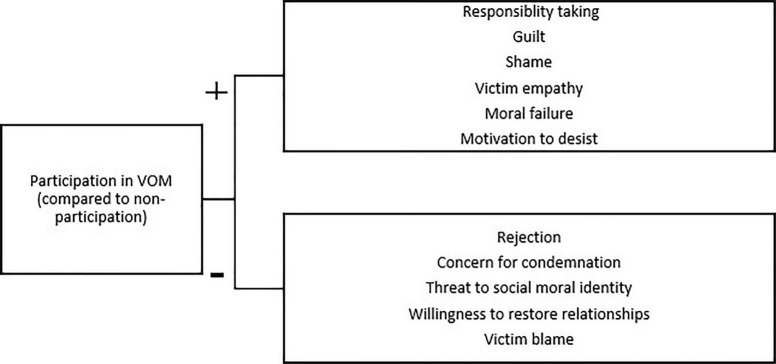
Overview of the hypothesized relations between participation in VOM and the dependent variables of interest.

In this research, we investigated the psychological effects of mediation on offenders. We compared offenders who participated in mediation with those who did not (most often because the victim declined). Figure 1 gives a visual overview of our hypotheses, based on the literature discussed above.

## Materials and Methods

### Victim-Offender Mediation Program in the Netherlands

We focused on mediation in criminal cases (*Mediation in Strafzaken* [MiS]) in the Netherlands. At the time of data collection for this study (October 2018–August 2020), MiS was a relatively new practice in the Netherlands. In 2017, after a pilot of 3 years, MiS was applied to all eleven criminal courts in the country. In MiS, a case is most often assigned to mediation after being referred from the public prosecutor. In a minority of MiS cases, the judge offers mediation during a court hearing. The public prosecutor or judge examines whether the victim and offender are open to mediation to deal with their criminal case. If so, the case is handed over to the mediation bureau at the criminal court. A mediation officer then contacts the offender and victim to confirm that they are both willing to participate. Sometimes, after receiving more information or having more time to think, parties decide to withdraw. When both parties agree to participate, the mediation officer assigns two mediators to the case and a meeting is scheduled. Before the offender and victim meet face-to-face, the mediators meet each party separately. In these pre-meetings, the mediator asks what happened, what the consequences were, and what the individual wants to achieve from mediation. The mediators always meet the offender first during this preparatory process. If the mediator thinks a conversation would be helpful for both parties, the actual mediation takes place. Most often, mediators plan the pre-meetings and mediation on the same day. In this study, all VOM meetings were face-to-face encounters between offenders and victims. During mediation, the victim and offender try to agree what the offender will do to repair the damage that was done. With permission from both parties, this agreement is communicated to the referring judge or public prosecutor, who take this agreement into account when deciding which punishment to impose. This means that the judge or public prosecutor has the final say in how the case will be solved (Claessen and Roelofs, 2020).

### Design

This research used a quasi-experimental pre- and posttest design to compare offenders who participated in VOM with those who did not. At the beginning of the VOM process, after referral to MiS, offenders were asked to fill in the first questionnaire. Six to eight weeks after the mediation dialogue between the parties had taken place, or 6–8 weeks after it was decided that mediation would not start, offenders were asked to fill in the second questionnaire. It was not possible to assign people randomly to the groups so we adopted a quasi-experimental approach. We aimed to compare these two groups while controlling for demographic and case-related variables. Participants who participated in mediation are referred to as the mediation group and participants who did not participate in mediation are referred to as the court group as their criminal case was handled by the conventional justice system.

### Participants

Ninety offenders participated in this study.^1^ After screening the data, four participants were excluded from further analyses. Two were excluded because of missing data (>50%) and extreme answers, one was excluded because they withdrew consent, and one was excluded because they scored neutral on every item and were manic at the time of the crime so did not remember what had happened. Of the remaining 86 participants, 64 were male (74%) and 22 were female (26%). The majority of these offenders were born in the Netherlands (*N* = 63, 73%) and almost half indicated high school (*N* = 39, 45%) as their highest level of education. A minority of offenders (*N* = 21, 24%) were religious. More than half of the participants were either married or had a stable relationship (62%) and 37% were single. Just over half of the participants lived together with their partner and/or children (*N* = 48, 56%) and 52% (*N* = 45) had children. Most offenders worked (*N* = 52, 61%). Others were unemployed (*N* = 23, 7%) because they were unfit for work or sick (*N* = 6/23), were retired (*N* = 6/23), were looking for a job (*N* = 7/23), or were addicted at the time of the offense (*N* = 1/23). Three offenders did not give a reason for their unemployment. Forty-seven offenders indicated that they were first-time offenders (55%). Table 1 summarizes the demographic characteristics.

**TABLE 1:** Overview of the demographic and case-specific variables of offenders (*N* = 86).

		*n*	%
Gender	Male	64	74
	Female	22	26
Highest education completed	Elementary school	2	2
	High school	39	45
	College	23	27
	Missing	22	26
Country of birth	Netherlands	63	73
	Other	5	6
	Missing	18	21
Religious	Yes	21	24
	No	48	56
	Missing	17	20
Living situation	With parents	13	15
	Living alone	24	28
	Living with partner/children	48	56
	Missing	1	1
Personal situation	No relationship	32	37
	In a relationship	53	62
	Missing	1	1
Being parent	Yes	47	55
	No	38	44
	Missing	1	1
Daily life activity	Student	9	11
	Unemployed	23	27
	Working	52	61
	Missing	1	1
Type of case	Personal	62	72
	Property	16	18
	Traffic	3	4
	Missing	5	6
District	Limburg	12	14
	Amsterdam	15	17
	Gelderland	5	6
	Overijssel	18	21
	Noord-Holland	13	15
	Rotterdam	2	2
	Den Haag	6	7
	Oost-Brabant	4	5
	Midden-Nederland	3	4
	Zeeland-West-Brabant	8	9
First time offender	No	18	21
	Yes	47	55
	Missing	21	24

Not all 86 participants completed a pretest and posttest. Thirty-seven participants filled in both the pretest and the posttest, whereas 35 participants completed the pretest only. Fourteen offenders only completed the posttest because their consent to participate arrived after the pretest had to be conducted. Fifty-five (64%) offenders participated in VOM and 31 (36%) did not because the victim declined (27/31, 87%) or because the offender declined (4/31, 13%).^2^ One offender refused to participate because he did not see the value of VOM. It was not clear why the remaining three offenders refused to participate. The victims were not asked why they refused to participate. Table 2 shows the number of participants per pre- and postmeasure. We asked offenders why they wanted to participate in VOM. In both groups, these reasons were to apologize or show regret, to talk things over with the victim, to rest the case more quickly without a judge or criminal prosecutor, to get on good terms with the victim, and to show the victim how wrong their behavior was. One offender in the mediation group said he participated in VOM to show his good side to the judge. There were no differences in reasons for participation between the two groups.

**TABLE 2:** Number of participants per measurement, distributed by group.

	Pretest	Posttest	Pre- and posttest	Total number of cases
Mediation group	20 (36%)	12 (22%)	23 (42%)	55
Court group	15 (48%)	2 (6%)	14 (45%)	31

The majority of offenders committed a personal offense (*N* = 62, 73%) – 48 of these were cases of violence or assault, eight were cases of threat, two were cases of stalking, one was an attempted homicide, one was a case of insult, one was a case of domestic violence, and one was a case of personal injury. Sixteen offenders committed a property offense – 11 of these were cases of vandalism, two were cases of theft, two were cases of trespassing, and one was a case of fraud. These cases were referred from ten different court jurisdictions in the Netherlands, but were not evenly distributed throughout the country. Most cases were from Limburg (*N* = 12, 14%), Amsterdam (*N* = 15, 17%), Overijssel (*N* = 18, 21%), and Noord-Holland (*N* = 13, 15%). However, all MiS bureaus had similar working procedures. Importantly, 11 cases (13%) were not administered by the MiS, but by a mediation practice in the Limburg region in the south of the Netherlands, with a criminal prosecutor leading the mediation rather than an independent mediator (Claessen et al., 2015). This difference in mediation practices was accounted for in the analysis.

### Dependent Variables

#### Responsibility Taking

All dependent measures were assessed using 5-point Likert scales or an alternative scale as indicated. For the 5-point Likert scale, participants rated to what extent they agreed with statements on a scale from strongly disagree (1) to strongly agree (5). If a different scale was used for a variable, this will be specifically mentioned. Otherwise, a 5-point Likert-scale is used. The scales and questionnaires were developed in 2017.

Responsibility taking was measured with four items. This construct measured to what extent offenders felt responsible for their offense leading to a police report and for the damage that their offense inflicted on the victims. Items included ‘I feel responsible for the offense’ and ‘It is my responsibility to restore the damage and pain that has been done to the victim.’ An exploratory factor analysis with one fixed factor indicated one underlying factor with an eigenvalue of 3.13 explaining 78% of the variance on the pretest and one underlying factor with an eigenvalue of 3.21 explaining 80% of the variance on the posttest. All items loaded high on this factor [Factor loadings (FLs) > 0.76].^3^ Reliability analyses indicated that the scale was reliable in the pretest and posttest (α = 0.91 and α = 0.92).

#### Feelings of Guilt About the Offense

Feeling guilty about the offense and toward the victim was measured with six items. These items were derived from the State Shame and Guilt Scale developed by Marshall and colleagues (as cited in Tilghman-Osborne, 2007). An exploratory factor analysis with one fixed factor on the pretest indicated one underlying factor with an eigenvalue of 4.25 explaining 71% of the variance on the pretest. The factor analysis with the posttest items showed two factors with an eigenvalue higher than one, but all items loaded high (FLs > 0.54) on the first factor with an eigenvalue of 3.50 explaining 58% of the variance. Items included ‘[When I think back about the offense] ‘I feel guilty’ and ‘I feel regret.’ The scale was reliable in the pretest and posttest (α = 0.91 and α = 0.85).

#### Shame

The items measuring appraisals and feelings of shame and rejection were adapted from those developed by Gausel et al. (2016). We aimed to distinguish between the two proposed appraisals (moral failure and concern about condemnation) and feelings (rejection and shame). With a factor analysis, we largely found the same distinctions between appraisal variables as Gausel et al. (2016) did. Concern about condemnation was measured with three items and perceived moral failure was measured with two items. An example item measuring concern about condemnation was ‘I am being rejected by others because of the offense’ and an example of an item measuring perceived moral failure was ‘What I did was wrong.’ Both appraisal scales were valid and reliable. An exploratory factor analysis with two fixed factors and two appraisal measure items entered simultaneously on the pretest and posttest indicated two underlying factors with an eigenvalue of 2.95 (concern about condemnation) and 1.46 (perceived moral failure), explaining 59 and 29% of the variance on the pretest and two underlying factors with an eigenvalue of 3.12 (concern about condemnation, all FLs > 0.94) and 1.34 (perceived moral failure, all FLs > 0.87), explaining 62 and 27% of the variance on the posttest. The concern about condemnation scale was reliable in the pretest and posttest (α = 0.94). The two items measuring appraisal for moral failure strongly correlated on the pretest [*r*(65) = 0.71, *p* < 0.005] and on the posttest [*r*(47) = 0.72, *p* < 0.005].

However, the factor analysis with the items that were supposed to measure rejection (three items) and shame (three items), showed one clear factor indicating rejection (eigenvalue of 3.64, explaining 61% of the variance), but not a second factor with an eigenvalue greater than 1 (just below 1). The item ‘I feel ashamed’ loaded weakly on the rejection factor but strongly on the second fixed factor, as intended. We therefore used this item as our measure of shame. The item ‘I feel small,’ which loaded high on both factors but was intended as an indicator of shame, was omitted from further analysis. The rejection scale therefore consisted of four items, including ‘When I think back about the offense, I feel alone’. With a Crohnbach’s α of 0.85 on both the pretest and posttest, the rejection scale was reliable.

#### Empathy

To correctly measure empathy, we intended to use items that measured both empathic concern and perspective taking. However, an exploratory factor analysis with two fixed factors on the pretest showed one factor with an eigenvalue of 5.62 explaining 70% of the variance. The same factor analysis on the posttest showed two factors with an eigenvalue of 5.16 and 1.10, explaining 78% of the variance. Looking at the factor loadings on the posttest, four items load high on factor one (FLs > 0.69). These items measured to what extent offenders could imagine how the victim felt, to what extent they were sorry for the victim, and to what extent they were able to put themselves in the victim’s shoes. These factors covered both the affective and cognitive scale. Therefore, it was decided to form one scale for empathy instead of distinguishing between empathic concern and perspective taking. The empathy scale was reliable in the pretest (α = 0.93) and the posttest (α = 0.92).^4^

#### Threat to Social Moral Identity

The perceived threat to the offender’s social moral identity was measured with four items adopted from Shnabel and Nadler (2008). An exploratory factor analysis with one fixed factor showed that all the items loaded high on the factor with an eigenvalue of 2.2, explaining 55% of the variance (FLs > 0.48), except for one item. These items measured to what extent the offender thought that others see them as unreliable and criminal because of the offense. A reliability test with the remaining three items showed that, after deleting the item measuring to what extent offenders thought that the victim perceived them as a bad person, the Cronbach’s α increased from 0.69 to 0.93. The two remaining items also correlated strongly with each other on the pretest [*r*(66) = 0.88, *p* < 0.001] and the posttest [*r*(47) = 0.94, *p* < 0.001], and therefore formed the scale ‘threat to moral identity.’

#### Restoring Damaged Relation With Victim

Two items measured to what extent the relationship with the victim was damaged by the offense. However, these items correlated negatively and weakly together [*r*(68) = --0.26, *p* = 0.032], so we analyzed them separately. One item was ‘If I run into the victim on the street right now, it would be very awkward,’ which was named relationship awkwardness. The other item was ‘At this moment, I would like to restore the relationship between the victim and myself’ and was named ‘relationship restoration^5^.’

#### Motivation to Desist

Motivation to desist measured to what extent offenders thought they would repeat their actions, whether they were able to prevent themselves from repeating their actions, and how likely they thought they were to repeat their behavior. These three items all measured one construct. An exploratory factor analysis with one fixed factor showed one factor with an eigenvalue of 2.08, explaining 69% of the variance on the pretest and one factor with an eigenvalue of 1.93, explaining 64% of the variance on the posttest (all FLs > 0.62). An example of an item measuring this variable was ‘I consider myself able to avoid repeating my negative behavior in the future.’ Both scales were reliable (pretest α = 0.77, posttest α = 0.73).

#### Victim Blame

We asked offenders to what extent they blamed the victim for what happened since we believe that this could interfere with other outcomes. Offenders who highly blame the victim for what happened might be less influenced by VOM^6^.

### Procedure

Data were collected between October 2018 and August 2020. When a case was referred to mediation and the mediation officer had contact with offenders, the mediation officer informed offenders about the research and asked if they wanted to participate in the study. In the first months of data collection the mediation officer asked offenders, during the first contact, to participate in the study. Multiple mediation officers indicated that offenders already received a high amount of information with that phone call and these calls are quite emotional. It was therefore not the right moment to also inform offenders about the study, in their view. In these first months, we did not recruit many participants, so changed our recruitment procedure. From May 2019 onward, an intern contacted offenders by phone, after a mediation officer had already made contact, to explain the study and invite them to participate. If offenders were willing to participate in the study, their name, email address, phone number, and case number were sent to the first author. The first author then sent the offender an email, explaining the research and including a link to the questionnaire. If the offenders did not fill in the questionnaire within 1 week, a reminder was sent by email. When offenders did not fill in the questionnaire after 2 weeks, they were called to ask if they were still willing to participate and reminded to fill in the questionnaire.

The mediation officer or the intern also informed the first author whether mediation would start or not. When mediation started, the first author was told when the face-to-face meeting would take place. Six to eight weeks after the mediation dialogue or after the researcher was informed that mediation would not start, a second questionnaire was sent to the offenders. The same reminders were sent after 1 and 2 weeks if the offenders did not complete the questionnaire.

The online-platform Qualtrics was used to distribute the survey. Personal links to the questionnaire were sent to offenders so the researcher could track which offenders had completed the questionnaire. When offenders opened the questionnaire, they first had to read an informed consent statement. The informed consent stated the aim of the research, how long it would take to participate, and that the study used a pretest and posttest. As an incentive to give informed consent, participants were told they could win one of five 15-euro gift cards. We explained how their data would be handled (that it would not be sent to others and that it would be stored in a secure digital environment) and that data would be made anonymous. Participants also allowed researchers to retrieve their judicial documentation after 2 years to see whether reoffences had been committed^7^. Offenders were allowed to withdraw their participation at any time without an explanation.

The questionnaire started with questions about demographics (gender; education; country of birth of the offender and their parents; religion; home situation; whether or not the offender had children and took care of these children; and the offender’s daily life activity). After demographics, the questionnaire measured the dependent variables on a 5-point Likert scale.

The posttest also started by obtaining informed consent and measuring demographics. Not all demographic variables were measured a second time since it was planned to measure these variables in the pretest only. Since this research was part of a larger project, other constructs were also measured on the posttest. These included how well prepared the participant was for mediation and how they experienced mediation or the justice process without mediation. Feelings of reintegrative shaming and stigmatization were also measured in the posttest. We also asked offenders if they had offered an apology and how it was received by the victim, and if the rules and norms they had violated during the offense were discussed in the mediation. Lastly, offenders were asked why they participated in mediation. These constructs were measured to determine which elements of the VOM process are responsible for the psychological outcomes. The focus of the present study was to determine whether VOM causes psychological changes in the offender. This study was approved by the Ethics Committee of the Faculty of Behavioural, Management and Social Sciences at the University of Twente (File number: 191033).

### Multiple Imputation

Some data were missing because participants dropped out or did not complete both a pretest and posttest. Listwise deletion would have resulted in a very small sample and low power, which might have biased the outcome (van Ginkel et al., 2020). Every variable contained some missing data; in total, 31% of the values were missing. Only 29 cases did not have missing data on the pretest and posttest. Most missing values were from posttest variables.

To maintain a sample of 86 participants, multiple imputation (MI) was applied, using SPSS statistics.^8^ With MI, complete versions of an incomplete dataset are formed, by replacing missing data points with a predicted value, based on a regression model plus a random error term (Little and Rubin, 1989; van Ginkel et al., 2020). This method has several advantages over listwise or pair wise deletion, but has not been used frequently in social sciences because of several misconceptions (van Ginkel et al., 2020). Some scholars have claimed that MI has disadvantages because it assumes that data are missing at random (Patrician, 2002) and it is very hard to determine if data are missing at random (Allison, 2000). Other researchers contradict this assumption of missing at randomness, as long as predictors that might explain missing at randomness are included when data is imputed (van Ginkel et al., 2020). What also should be taken into account when using MI, is to not accept imputations that are very different from the observed data (Van Buuren, 2018). We decided to use MI in this study because listwise and pairwise deletion could also lead to bias if data are not missing at random (van Ginkel et al., 2020) and reduce the sample size and statistical power.

Using default settings in IBM SPSS statistics 25, MI was used to estimate and fill in missing data. All measured variables with missing data were imputed and used as predictors. The variables that did not have missing data (gender and mediation practice) were added as predictors for the imputation of other variables. The minimum and maximum constraint were set according to the 5-point Likert-scale and were rounded to the nearest integer. Considering the amount of missing data, 30 imputations were done and pooled outcomes were used in hypotheses testing (White et al., 2011). Because SPSS bases imputation on the whole dataset and the data consist of two different groups (court group and mediation group), two separate imputations were conducted: one on a dataset containing the measures of the mediation group only and one on a dataset containing the measures of the court group only. Once the imputation was done, the datasets were added together.^9^ Table 3 shows the pooled means and standard deviations (SD) of the original data and the MI data.^10^ The means and SDs of the original data were almost the same as those of the imputed data (largest difference in means is 0.1). Since the imputed dataset shows the same pattern as the original data and provides a complete data set with 86 participants, these data were used to test the proposed hypotheses.

**TABLE 3:** Means, standard deviations, and number of participants per variable per group.

	*M* (*SD*) Original data (*n* = 16–42)				*M* (*SD*) Imputed data (*n* = 86)			
	**Court**		**Mediation**		**Court**		**Mediation**	
	**Pretest**	**Posttest**	**Pretest**	**Posttest**	**Pretest**	**Posttest**	**Pretest**	**Posttest**
	
Responsibility taking	2.4 (1.1)	2.3 (1.2)	2.6 (1.2)	2.9 (1.0)	2.4 (1.1)	2.3 (1.0)	2.6 (0.92)	2.9 (1.1)
Guilt	2.6 (1.2)	2.4 (0.94)	3.1 (1.0)	3.0 (0.91)	2.7 (1.2)	2.4 (0.76)	3.1 (0.95)	3.0 (0.80)
Shame (one item)	2.5 (1.4)	2.1 (1.1)	2.9 (1.4)	2.7 (1.4)	2.5 (1.4)	2.0 (1.2)	2.8 (1.5)	2.7 (1.4)
Moral failure	2.5 (1.3)	2.1 (1.1)	2.8 (1.2)	2.8 (1.1)	2.5 (1.3)	2.2 (0.99)	2.9 (1.2)	2.8 (1.0)
Concern about condemnation	1.8 (1.1)	1.7 (0.83)	2.1 (1.1)	1.6 (0.79)	1.8 (1.1)	1.7 (0.66)	2.2 (1.1)	1.7 (0.70)
Feeling of rejection	2.0 (0.88)	2.0 (0.85)	2.3 (0.96)	2.0 (0.95)	2.0 (0.71)	2.1 (0.69)	2.3 (0.89)	2.1 (0.84)
Empathy	2.7 (1.2)	2.6 (1.2)	2.9 (1.2)	3.3 (1.1)	2.7 (1.2)	2.6 (0.99)	3.0 (1.1)	3.2 (0.97)
Victim blame (one item)	3.9 (1.3)	3.9 (1.2)	4.1 (1.2)	3.8 (1.5)	3.8 (1.3)	3.8 (1.2)	4.0 (1.3)	3.7 (1.5)
Threat to moral identity	2.0 (1.1)	1.6 (0.90)	1.9 (1.0)	1.7 (0.91)	2.0 (1.0)	1.7 (0.80)	1.9 (0.98)	1.8 (0.86)
Awkwardness (one item	2.6 (1.4)	2.9 (1.4)	3.3 (1.3)	2.2 (1.1)	2.6 (1.4)	2.9 (1.5)	3.3 (1.3)	2.2 (1.2)
Restore victim relationship (one item)	3.2 (1.3)	3.2 (1.2)	3.5 (1.0)	2.9 (1.1)	3.2 (1.4)	3.2 (1.4)	3.5 (1.1)	2.9 (1.3)
Motivation to desist	3.9 (0.73)	3.9 (1.2)	4.1 (1.0)	4.2 (0.71)	3.9 (0.72)	4.0 (0.85)	4.0 (0.92)	4.1 (0.69)

## Results

### Descriptive Statistics

The mean values of the whole sample (Table 3) showed that offenders were not concerned about condemnation or did not feel rejected at the pretest (*M* = 1.8–2.3) or posttest (*M* = 1.7–2.1). They were quite neutral on their feelings of shame (*M* = 2.1–2.9) and on their willingness to restore the relationship with the victim (*M* = 2.9–3.5). The offenders scored low to neutral on awkwardness to meet the victim (*M* = 2.2–3.3) but scored high on blaming the victim in both the pretest (*M* = 3.8 and *M* = 4.0, respectively) and the posttest (*M* = 3.8 and *M* = 3.7, respectively). The offenders did not experience a high threat to their social moral identity (*M* < 2.0) and already had a high motivation to desist from crime on the pretest (court group *M* = 3.9 and mediation group *M* = 4.0). This indicates that our participants were not afraid of being rejected or perceived as an outcast by others.

The pretest scores between the two groups were similar for all variables, except for awkwardness to meet the victim where participants in the mediation group scored an average 0.7 points higher than participants in the court group did. However, more differences between the groups emerged on the posttest (Table 3). We observed differences in scores on taking responsibility, feeling guilt, feeling shame, appraising moral failure, and empathizing with the victim. The mediation group scored consistently higher on these variables in the posttest than the court group did. Interestingly, responsibility taking and victim empathy increased from the pretest to the posttest in the mediation group, but not in the court group. There were also differences between the two groups in scores on awkwardness when meeting the victim in the posttest. However, in contrast to the pretest scores where the mediation group scored higher, the mediation group scored lower than the court group in the posttest.

We observed that guilt, moral failure, rejection, shame, concern about condemnation, and responsibility taking correlated positively with each other but that there was no correlation between concern about condemnation and responsibility taking (Table 4). Interestingly, lower scores on the abovementioned variables (except for rejection) were related to more victim blame. Furthermore, wanting to restore the relationship with the victim correlated positively with empathy but negatively with victim blame. This indicates that wanting to restore the relationship with the victim is related to stronger victim empathy and lower victim blame.

**TABLE 4:** Pearson correlations between the dependent variables on the posttest.

	1	2	3	4	5	6	7	8	9	10	11	12
(1) Responsibility taking	–											
(2) Guilt	0.601**	–										
(3) Moral failure	0.593**	0.667**	–									
(4) Concern about condemnation	0.197	0.418**	0.276*	–								
(5) Rejection	0.243*	0.333**	0.279*	0.519**	–							
(6) Shame	0.461*	0.631**	0.519**	0.240*	0.309**	–						
(7) Empathy	0.608**	0.708**	0.561**	0.336**	0.332**	0.547**	–					
(8) Victim blame	–0.303**	–0.312**	–0.304**	–0.245*	–0.080	–0.309*	–0.422**	–				
(9) Threat to social moral identity	–0.015	0.250*	0.155	0.636**	0.418**	0.181	0.153	–0.074	–			
(10) Awkwardness	–0.168	–0.012	–0.127	0.169	0.130	0.082	–0.127	0.186	0.249*	–		
(11) Restore victim relation	0.195	0.165	0.130	0.043	0.047	0.162	0.258*	–0.241*	–0.009	–0.032	–	
(12) Desisting	0.305**	0.450**	0.382**	0.186	0.275*	0.358**	0.435**	–0.184	0.079	–0.041	0.197	–

*Pearson correlation p < 0.05.

**Pearson correlation p < 0.01.

*All correlations in bold are significant at a p-value < 0.05 or <0.01.*

### Hypothesis Testing

Multiple regression analyses were used to test whether mediation (versus no mediation) is associated with differences in the dependent variables. Using a plot of the standardized residuals and the standardized predicted values, we checked for linearity and homoscedasticity. A histogram of the residuals was used to check the assumption of normality. These assumptions were met.

In these regression analyses, the mediation group was coded as 1 and the court group was coded as 0. We controlled for the pretest scores of the dependent (posttest) variable, which controlled for any differences in pretest scores between the mediation and court group. We used an Anova test to determine whether pretest scores were different between the two groups. One significant difference was found and these results are available on request. To eliminate self-selection bias, we controlled for pretest scores in the analyses. We also controlled for all demographic and case-related background variables that were assessed in this study: age, gender, type of case (dummy coded), highest finished education (dummy coded), country of birth (of the offender and their parents), religion, living situation (dummy coded), personal status, having children or not, daily life activity (dummy coded), and if someone was a first offender or not.

In line with our expectations, the regression analyses showed a significant effect of participation in VOM on responsibility taking (*B* = 0.59, *t* = 2.45, *p* = 0.014), guilt (*B* = 0.44, *t* = 2.15, *p* = 0.031), appraisal for moral failure (*B* = 0.59, *t* = 2.12, *p* = 0.035), shame (*B* = 0.74, *t* = 2.16, *p* = 0.031), and victim empathy (*B* = 0.54, *t* = 2.25, *p* = 0.025). Offenders who had participated in mediation scored significantly higher on variables in the posttest than offenders who did not participate in VOM did. Also, a significant effect was found for awkwardness (*B* = –0.86, *t* = –0.26, *p* = 0.011); offenders who participated in VOM thought that it would be less awkward to meet the victim in daily life afterward than offenders who did not participate did. Except for pretest scores, other background variables did not significantly affect the dependent variables (Table 5). As shown in Table 6, no other significant effects were found. These regression analyses partly confirmed our hypotheses. Eliminating the four offenders who were not willing to participate in mediation from the model did not yield different outcomes.

**TABLE 5:** Pooled regression coefficients for the significant effects, with type of group as predictor, controlled for demographic and case-related variables.

	Responsibility taking				Guilt				Appraisal for moral failure				Shame				Empathy				Awkwardness			
	*B*	*SE*	*t*	*p*	*B*	*SE*	*t*	*p*	*B*	*SE*	*t*	*p*	*B*	*SE*	*t*	*p*	*B*	*SE*	*t*	*p*	*B*	*SE*	*t*	*p*
Age	0.004	0.01	0.43	0.668	0.000	0.01	0.04	0.972	<0.005	0.01	0.001	0.999	0.01	0.02	0.61	0.540	–0.001	0.01	–0.10	0.921	<0.005	0.01	–0.02	0.987
Case type (property = 0)	0.09	0.54	0.17	0.867	0.21	0.46	0.46	0.645	0.04	0.62	0.06	0.951	0.47	0.78	0.61	0.546	–0.03	0.56	–0.05	0.957	0.06	0.69	0.09	0.930
Case type (personal = 0)	0.20	0.57	0.35	0.725	0.17	0.49	0.34	0.735	0.08	0.66	0.12	0.906	0.39	0.87	0.45	0.650	0.14	0.60	0.24	0.815	0.03	0.77	0.04	0.967
Mediation practice (no mediation = 0)	0.07	0.35	0.20	0.840	0.12	0.30	0.40	0.690	–0.05	0.39	–0.14	0.893	–0.11	0.53	–0.22	0.829	0.27	0.37	0.73	0.464	0.26	0.48	0.54	0.590
Gender (male = 0)	0.07	0.25	0.27	0.791	0.07	0.21	0.34	0.732	0.13	0.27	0.49	0.625	0.17	0.36	0.47	0.641	0.31	0.25	1.23	0.220	0.08	0.32	0.24	0.812
Education (preschool = 0)	0.28	0.79	0.35	0.727	0.17	0.65	0.26	0.165	0.15	0.74	0.20	0.845	0.06	0.382	0.15	0.881	0.02	0.86	0.03	0.980	0.12	0.87	0.13	0.895
Education (high school = 0)	–0.02	0.24	–0.06	0.949	0.03	0.21	0.13	0.894	0.01	0.27	0.05	0.964	0.08	0.96	0.08	0.934	–0.02	0.25	–0.08	0.937	–0.02	0.31	–0.07	0.948
Country of birth (Netherlands = 0)	–0.16	0.63	–0.26	0.796	–0.18	0.50	–0.37	0.713	–0.13	0.66	–0.19	0.849	–0.29	0.81	–0.36	0.722	–0.19	0.63	–0.31	0.758	0.13	0.66	0.19	0.848
Country of birth father (Netherlands = 0)	0.05	0.52	0.09	0.925	0.01	0.40	–0.02	0.988	–0.02	0.53	–0.03	0.976	0.02	0.62	0.04	0.972	0.03	0.52	0.06	0.951	0.03	0.59	0.05	0.962
Country of birth mother (Netherlands = 0)	0.08	0.57	0.15	0.883	–0.001	0.51	–0.002	0.999	–0.07	0.59	–0.12	0.906	0.21	0.73	0.29	0.771	–0.12	0.61	–0.19	0.849	0.27	0.63	0.43	0.665
Religious (no = 0)	0.02	0.28	0.09	0.932	–0.03	0.22	–0.14	0.889	0.130	0.30	0.43	0.665	–0.29	0.37	–0.69	0.488	–0.13	0.28	–0.46	0.647	–0.03	0.34	–0.09	0.929
Living situation (with parents = 0)	–0.18	0.50	–0.36	0.723	–0.003	0.41	–0.007	0.995	–0.28	0.54	–0.52	0.605	0.02	0.75	0.03	0.978	–0.25	0.54	–0.56	0.650	0.17	0.65	0.26	0.797
Living situation (alone = 0)	0.25	0.39	0.64	0.523	0.28	0.34	0.84	0.404	–0.01	0.45	–0.02	0.984	0.18	0.58	0.31	0.760	–0.01	0.373	–0.02	0.984	0.44	0.52	0.86	0.392
Relationship (no = 0)	0.11	0.38	0.30	0.765	0.05	0.30	0.15	0.881	0.25	0.42	0.60	0.55	0.19	0.49	0.38	0.703	0.13	0.35	0.38	0.705	0.10	0.44	0.22	0.824
Parent (no = 0)	–0.12	0.30	–0.40	0.686	–0.02	0.25	–0.08	0.935	–0.001	0.31	–0.004	0.996	0.01	0.40	0.02	0.987	0.04	0.29	0.12	0.901	–0.43	0.37	–1.18	0.238
Daily life activity (student = 0)	–0.25	0.58	–0.43	0.667	0.06	0.46	0.13	0.899	0.04	0.58	0.07	0.941	–0.08	0.79	–0.10	0.917	–0.03	0.51	–0.06	0.950	0.05	0.71	0.07	0.941
Daily life activity (employed = 0)	–0.02	0.27	–0.06	0.956	0.17	0.22	0.78	0.44	0.09	0.29	0.30	0.762	0.04	0.39	0.09	0.926	–0.07	0.26	–0.25	0.800	–0.67	0.36	–0.74	0.461
First offender (yes = 0)	0.22	0.30	0.73	0.468	0.16	0.26	0.62	0.534	0.11	0.33	0.33	0.745	–0.04	0.41	–0.09	0.932	0.21	0.29	0.72	0.475	–0.08	0.39	–0.20	0.844
Score on pretest	0.32	0.12	2.68	**0.008**	0.26	0.10	2.52	**0.012**	0.21	0.13	1.660	0.098	0.26	0.14	1.94	0.054	0.38	0.11	3.52	** < 0.001**	0.31	0.13	0.24	**0.020**
Type of group (court group = 0)	0.59	0.24	2.45	**0.014**	0.44	0.21	2.15	**0.031**	0.59	0.28	2.12	**0.035**	0.74	0.34	2.16	**0.031**	0.54	0.24	2.25	**0.025**	–0.86	0.34	–0.26	**0.011**

*N = 86.*

*Significant outcomes are given in bold.*

**TABLE 6:** Pooled regression coefficients for type of group as predictor, controlled for demographic and case-related variables for the non-significant outcomes (*N* = 86).

	*B*	*SE*	*t*	*p*
Concern about condemnation	–0.23	0.18	–1.30	0.196
Rejection	–0.17	0.22	–0.79	0.429
Threat to social identity	0.06	0.21	0.27	0.786
Need to restore victim relation	–0.41	0.38	–1.10	0.275
Motivation to desist	0.04	0.20	0.18	0.858
Victim blame	–0.24	0.34	–0.69	0.493

## Discussion

The main goal of this research was to examine whether VOM changes psychological outcome variables in offenders. Our findings offer some support for the proposed hypotheses. Analyses showed that offenders who participated in VOM took more responsibility 6–8 weeks after VOM than offenders who did not participate in VOM did. This is in accordance with previous qualitative research, which showed that hearing the impact of the offense from the victim during VOM affects the amount of regret and responsibility the offender feels (Choi et al., 2011; Miller and Hefner, 2015). Pabsdorff et al. (2011) also indicated that VOM focuses on how offenders can take responsibility for their actions, which may explain our findings.

Offenders who participated in VOM had stronger feelings of guilt than offenders who did not participate in VOM did. However, this effect does not seem to be due to the VOM process increasing feelings of guilt, but was rather due to consolidation of guilt during and after the VOM process. That is, feelings of guilt decreased compared with the premeasure in the court group but not in the mediation group. The same was true for shame; offenders who participated in VOM felt more ashamed afterward than offenders who did not participate did, probably because feelings of shame were consolidated during VOM. Marsh and Maruna (2016) also argued that offenders might experience more guilt and shame after VOM, because they are more aware of the impact of their crimes. According to Gausel et al. (2016), feelings of shame are related to the appraisal of a moral failure, which is in agreement with our findings. Offenders who participated in VOM showed a higher appraisal of moral failure than offenders who did not participate in VOM did. By understanding the effects of their behavior, offenders might realize that this behavior was not in accordance with the moral rules of society. As a consequence, offenders might experience a higher moral failure and become more self-critical of their behavior. Participating in VOM did not affect concern about condemnation or rejection, possibly because the participants scored relatively low on these variables in the pretest. Nevertheless, these scores did not increase, which suggests that VOM does not have a stigmatizing effect.

Offenders also showed more victim empathy after VOM, in agreement with previous findings. Lauwaert and Aertsen (2016) argued that VOM helps offenders to understand their victim’s point of view and empathize with their victim (Miller and Hefner, 2015; Meléndez, 2020b).

In our study, participating in VOM had no effect on the perceived threat to social moral identity or on wanting to restore the relationship with the victim. This could be because our participants did not feel that their social moral identity was threatened and did not want to restore their relationship with the victim before VOM started. VOM might have had more of an effect if the offender had already felt this threat and had this willingness.

It is important to note that we did not measure the variables immediately after VOM, but rather 6–8 weeks later. This indicates that VOM has an impact for at least 6–8 weeks. However, it is unclear how sustainable these effects are in the long run. Reoffending is typically investigated after more than 1 year (Hansen and Umbreit, 2018; Jonas-van Dijk et al., 2020), so it remains unclear whether the psychological changes we observed are relevant to reoffending behavior. It would be interesting to adopt a longitudinal research design in a future study and administer an additional questionnaire to offenders months or a few years after the mediation encounter and to determine whether any reoffences have occurred. This would uncover whether the psychological changes observed after the mediation encounter are related to reoffending behavior. However, the dropout rates that we and others have observed (Cleven et al., 2015) indicate that achieving an adequate sample size would be challenging in a longitudinal research set up.

Another strength of our study is that most outcome variables did not differ significantly between the two groups when VOM started and that any differences were controlled for in the analyses. Since both groups almost entirely consisted of offenders who were willing to participate, the effects we observed are most probably due to the VOM process and not due to self-selection bias. Motivations to participate in VOM were also comparable for offenders who participated in VOM and those who did not. However, the outcomes we observed may have been due to the punishment imposed on the offender. After mediation, the public prosecutor or judge decides which, if any, punishment to impose. The agreement made between the victim and offenders during mediation may have resolved what happened. This means that, in some cases, offenders in the mediation group might not have received a punishment whereas offenders in the court group did. The effect of punishment on the outcome variables and reoffending should be examined in future studies. The reasons the victims rejected participation in VOM may also explain our results. We did not ask the victims to explain why they declined VOM, but this information could have said something about the offender. For example, the victim might have thought that the intentions or motivations of the offender to participate were insincere. This should also be investigated in future research.

We did not observe the expected effects of VOM on all outcome variables. One explanation could be that, in both groups, offenders highly blamed the victim for what happened and the VOM process did not lower this victim blame. This blame might have influenced the effectiveness of the VOM program. Another explanation could be the small sample size, which negatively affected the power of the study. We wanted to include more participants but this was not possible because of time limits and restrictions related to the COVID-19 pandemic. Because of the COVID-19 pandemic, organization of new mediation encounters was restricted between March 2020 and September 2020. There was also a high dropout rate, especially on the posttest. It was challenging to motivate offenders to participate in both the pretest and posttest. This problem with dropout has also been reported in similar studies (Meléndez, 2015). We used MI to account for dropout, which means that conclusions were based on data that were partly estimated by a statistical program. However, the original dataset showed highly similar patterns to the imputed dataset, suggesting that our conclusions are reliable.

Another explanation for not observing stronger effects of VOM on the outcome variables, could be that the single 1-h face-to-face conversation between the victim and offender was not enough to affect the offenders and their relation with their victim. Umbreit (1994) also claimed that a VOM program should not be expected to elicit major effects. Milder effects are more common in these types of programs, which means there are limits on what VOM can achieve (Daly, 2017). VOM can maximize a desisting process that has already begun (Lauwaert and Aertsen, 2016). We also observed that offenders in this study were already highly motivated to desist from crime. As Woolpert noted (as cited in Wyrick and Costanzo, 1999) “One should not expect exposure to a victim offender reconciliation program (VORP) by itself to have a major impact on offenders, whose lives are typically beset by countless personal problems and repeated instances of failure and antisocial behavior. For some, participating in a VORP may be the first socially approved act they have successfully performed. Any program that shows evidence of even slight improvement in the outlook and conduct of offenders, however, is welcome” (p. 255).

Every VOM program is unique and we did not examine which elements of the program were responsible for the effects we observed, which makes it hard to generalize the outcomes. However, our VOM program is similar to the four-step VOM process described by Hansen and Umbreit (2018), so we believe that our findings can be generalized to other VOM programs that focus on the conversation between the victim and the offender. Future research should combine observational data with data from a pre- and postmeasure questionnaire to examine which elements of a VOM program are responsible for psychological change in offenders. One element could be the mediation style, which may differ between mediators. The mediator has an important role during the VOM process because they can help the offender with the desistance process, for example (Lauwaert and Aertsen, 2016). Therefore, it might be worth examining how the mediation style affects the attitude and behavior of the offender.

Although this study had some limitations, it revealed important patterns of VOM participation on offenders. To our knowledge, this is one of the first studies to examine the psychological effects of VOM on offenders using a pretest and posttest and with a control group of offenders who were willing to participate in VOM but did not – these data have been missing in other studies into restorative justice practices (Elbers et al., 2020). However, the effects we observed are modest and should be interpreted with caution because of the small sample size and the use of MI. The small sample size might heighten the change on small variations in offender characteristics, which might have influenced the outcomes. However, we controlled for background variables and considered how they may have influenced the results. Nevertheless, the findings suggest that VOM can foster conducive feelings and cognitions among offenders in terms of responsibility taking, feelings of shame, perceived moral failure, feelings of guilt, and victim empathy. We also observed that VOM makes offenders more aware of the impact of their crime, which might explain these psychological changes. One VOM meeting might not move mountains, but can elicit psychological changes in offenders that may reduce the risk of reoffending (Latimer et al., 2005; Jonas-van Dijk et al., 2020). Future studies should examine whether these psychological changes reduce the risk of reoffending.

## Data Availability Statement

The anonymous dataset is available on request, please contact JJ via j.jonasvandijk@utwente.nl.

## Ethics Statement

The studies involving human participants were reviewed and approved by the Ethics Committee of the Faculty of Behavioural, Management and Social Sciences of the University of Twente (File number: 191033). The patients/participants provided their written informed consent to participate in this study.

## Author Contributions

All authors contributed to the design of the study setup. JJ collected the data and analyzed the data with help from SZ. JJ wrote the manuscript. SZ, JC, and HN edited the manuscript.

## Conflict of Interest

The authors declare that the research was conducted in the absence of any commercial or financial relationships that could be construed as a potential conflict of interest.

## Publisher’s Note

All claims expressed in this article are solely those of the authors and do not necessarily represent those of their affiliated organizations, or those of the publisher, the editors and the reviewers. Any product that may be evaluated in this article, or claim that may be made by its manufacturer, is not guaranteed or endorsed by the publisher.

## Footnotes

1 Participants were asked to participate in the study after referral to the mediation process, but before the mediation process started. For pragmatic reasons, we consistently refer to ‘offenders’ in the context of this research on VOM, although we are aware that within a criminal case an individual is only an offender when proven guilty.

2 Analysis in this research were also done while leaving out the four offenders who were not willing to participate and this yielded the same patterns of results.

3 The cut off score for factor loadings (FLs) of the exploratory factor analyses (EFA) was set at 0.4.

4 Three other items were included to measure empathic concern and perspective taking. However, these three items did not load on the empathy factor and measure perceived emotions. Analysis of this factor did not yield any effects.

5 Initially we also examined to what extent offenders wanted to restore the relationship with family, friends, the community and the self. However, for validity reasons, we decided to not include that scale. In addition, analysis did not show any effects.

6 This item was part of scale to measure neutralizing. However, a factor analysis showed that this item did not load high on the neutralizing scale. Since analysis showed no differences between the two groups on neutralizing and the sample showed no signs of neutralizing (*M* = 1.7), we decided to not further report this variable.

7 Data on reoffending will be used in future research.

8 *T*-tests and chi-square tests were done to examine selective drop out. Drop out was examined for seventeen variables, of which three were significant. In analyses it will therefore be controlled for background variables. Details on these tests are available on request.

9 Imputations were also done for the complete dataset. However, the means of these imputed data were deviant from the original data. It was therefore decided to impute the data in two different datasets.

10 The pooled SDs were manually obtained from the standard error of the mean, multiplied by the square root of the sample size.
